# Dimensions of control and their relation to disordered eating behaviours and obsessive-compulsive symptoms

**DOI:** 10.1186/s40337-016-0104-4

**Published:** 2016-05-03

**Authors:** Franzisca V. Froreich, Lenny R. Vartanian, Jessica R. Grisham, Stephen W. Touyz

**Affiliations:** School of Psychology, UNSW Australia, Sydney, New South Wales 2052 Australia; School of Psychology, University of Sydney, Sydney, 2006 New South Wales Australia

**Keywords:** Assessment of control, Disordered eating, Fear of losing self-control, Ineffectiveness, Obsessive-compulsive symptoms

## Abstract

**Background:**

Issues of personal control have been proposed to play a central role in the aetiology and maintenance of eating disorders. Empirical evidence supporting this relationship is inconsistent, partly due to the multiplicity of constructs used to define “control”. This study compares six commonly used measures of control with the aim of determining which operationalisation of control is most centrally relevant to eating pathology. Given the high level of comorbidity between eating disorders and obsessive-compulsive disorder and the potentially common risk/maintenance factors for the two disorders, we also examine the relationship between control and obsessive-compulsive symptomatology.

**Methods:**

Female community participants (*N* = 175) completed self-report measures of control, eating disorder pathology and obsessive-compulsive symptoms.

**Results:**

Multivariate analyses of variance indicated significant differences between individuals with high vs. low levels of psychopathology on most of the measures of control. Using regression analyses, we found that a sense of ineffectiveness and fear of losing self-control were the only significant independent predictors of eating pathology, and fear of losing self-control was the most significant predictor of obsessive-compulsive symptoms.

**Conclusions:**

This study highlights the importance of issues of control, particularly feelings of ineffectiveness and fear of losing self-control, in eating disorder symptoms. Furthermore, our findings suggest that there may be a similar underlying fear of losing self-control among individuals who engage in disordered eating and obsessive-compulsive behaviours. Thus, ineffectiveness and fear of losing self-control are two dimensions that are important to consider in maintenance and treatment models of disordered eating behaviours.

## Background

Issues of personal control have been proposed to play a central role in the aetiology and maintenance of eating disorders (ED), in particular Anorexia Nervosa (AN). For example, Bruch defined AN as a “struggle for control, for a sense of identity, competence, and effectiveness” ([1] p. 251). In her view, AN symptomatology can be understood as a desperate attempt to compensate for an underlying sense of ineffectiveness and lack of control experienced in the rest of the individual’s life. Similarly, Slade hypothesised that control in weight loss enables individuals with AN to avoid negative affect associated with general life dissatisfaction and interpersonal problems. Control over eating becomes the primary focus because it is perceived as “successful behaviour in the context of perceived failure in all other areas of functioning” ([2] p. 173). Several other authors have proposed models of AN maintenance that emphasise the role of control within the disorder, noting that individuals use control over weight and shape as an index of overall self-control and self-worth [3], that they control their environment, especially close family members through their illness [4], and that the condition is reinforced through the individual’s intense fear of loss of control [5]. Although the representation of control within these accounts do vary to some extent, they share the underlying premise that obsessive restriction over food and weight represent strategies to cope with generalised feelings of perceived lack of control. In the absence of adaptive personal control strategies, the individual may be driven to enact ritualistic body control as an auxiliary control mechanism.

Although most research on psychological control has been conducted with AN patients, those studies that have included individuals with Bulimia Nervosa (BN) have generally found no significant difference between AN and BN on the basis of control variables [6–8]. Slade [2] highlighted that a subset of individuals who develop BN have transitioned from AN. He argued that, at least for these patients, bulimic behaviour is an attempted alternative method of weight control that does not involve self-starvation and therefore bypasses direct confrontation with other people. Indeed, longitudinal research finds that 20–50 % of individuals with AN will develop BN over time [9–11], which speaks to the instability of ED diagnoses and why it may be difficult to find risk factors that differentiate between ED syndromes. Thus, control issues may be relevant not only for AN but across the ED spectrum.

Studies using qualitative methods provide support for the notion that perceived lack of control is a major contributing factor to the development of ED (see [12] for an example). Many patients recall starting dieting at a time when they perceived life to be chaotic and out of control. In this context, controlling one’s food intake is seen as the “solution”. As one patient described: “When I started changing my eating habits it was… because I didn’t feel in control of my life or of myself. Controlling what I ate was one way of controlling at least part of my life… I felt that if I could control what went in and out and how much exercise I did then I could control other things in my life” ([13] p. 16).

Despite the strong theoretical and qualitative link between issues of control and ED, evidence from quantitative studies is limited and inconsistent. One difficulty in this area of research is that there is considerable heterogeneity in how the construct “control” is defined (see [14] for a review). A cursory review of research in the area reveals a large number of terms that have been used to conceptualise control, including *sense of control*, *locus of control*, *fear of losing control*, *desire for control*, *mastery*, and *ineffectiveness*. Although these terms may be interrelated and partially overlapping, they have been found to produce different results in relation to ED, thus complicating our understanding of the link between ED and control. For example, a sense of *ineffectiveness*, which reflects feelings of deficient control and worthlessness, and a low *sense of control* have consistently been found to influence risk and maintenance of ED [15–17]. In fact, Ineffectiveness is one of the subscales of the Eating Disorders Inventory [18], a measure of ED attitudes and behaviours that is widely used in research settings and clinical practice. Furthermore, in one study that compared multiple measures of control, *fear of losing self-control* was the most significant predictor of ED symptomatology [7] but this finding still awaits replication.

In contrast to the constructs described above that have shown consistent results across studies, other constructs have received inconsistent support. Studies assessing *locus of control,* which refers to an individual’s belief about the source of control over reinforcement [19], have found that women who report engaging in disordered eating behaviours show greater externality [20], greater internality [21], and even no difference compared to healthy controls [22]. Similarly, studies exploring *desire for control* have found that ED patients have lower [7] or similar levels [16] of motivation to control the events in their lives compared to healthy controls.

In short, control appears to be a concept of importance in relation to ED but some of its dimensions are more consistently related to ED symptoms than are others. Determining which operationalisation of control is most centrally relevant to ED is important, not only because it will aid with the accumulation of research findings, but also because it could help clinicians understand which specific aspects of control (if any) need to be assessed and perhaps addressed in treatment. Past research in this domain has predominantly examined a single aspect of control in relation to ED symptomatology [20, 23], with only a few studies comparing up to three dimensions of control [7, 21]. Thus, the primary aim of the current study was to build on previous research by examining a broader range of constructs in order to determine which form (or forms) of control are most strongly associated with eating pathology. Specifically, this study compares six constructs commonly used in the eating-disorder literature to assess issues of control: locus of control, sense of control, fear of losing self-control, desire for control, sense of mastery, and ineffectiveness. We predicted that women who score high on a measure of disordered eating would show a weaker sense of personal control than would women who score low on the measure of disordered eating. We also assessed which specific measure(s) would emerge as the strongest predictor(s) of ED symptomatology but, given the inconsistencies in previous research, no firm predictions were made in this case.

A secondary aim of this study was to explore the relationship between control and obsessive-compulsive symptomatology. Obsessive-compulsive disorder (OCD) and ED are highly comorbid [24] and a number of authors have suggested that there may be common risk factors and/or shared mechanisms for maintenance of the two disorders [25, 26]. Similar to ED, phenomenological descriptions of OCD have often highlighted the role of control within the disorder. For example, individuals exhibiting OCD symptoms have been noted to “dislike spontaneity and prefer safety and predictability in order to appease their strong need for control over the environment” ([27] p. 756). Furthermore, OCD symptoms have been associated with a lower sense of control over the self and the environment, and this discrepancy between desire for control and perceived control is thought to motivate compulsive symptoms [28]. In these ways, it appears that both conditions, OCD and ED, may have functionally similar clinical presentations; that is, both represent attempts to reassert control, albeit through different means. In light of these apparent similarities between ED and OCD, we examined whether the constructs of control that are relevant to ED are also related to OCD symptoms. Because of the lack of research in this domain, no firm predictions were made.

## Methods

### Participants

Data were collected through Amazon Mechanical Turk (MTurk), a crowdsourcing website. Female MTurk registered users residing in the United States between the ages of 18 and 40 were eligible to participate. Participants were excluded if they provided incomplete data or if they failed any of the validity checks that were included in the survey. Valid and usable data were available for 175 female participants, aged 19–40 years (*M* = 30.25, *SD* = 5.7). Their mean body mass index (BMI [kg/m^2^], based on self-reported height and weight) was 22.95 (*SD* = 6.81). Participants identified primarily as White/Caucasian (*n* = 138; 78.9 %), followed by African-American (*n* = 15; 8.6 %), Hispanic (*n* = 9; 5.1 %), Asian (*n* = 6; 3.4 %), and “other” (*n* = 7; 4.0 %).

### Materials and procedure

Participants signed up for an online study that was described as an investigation of peoples’ experiences of control in their lives. After reading an introductory information page and indicating their consent, participants completed the questionnaires described below. Control-related questionnaires were presented first, in randomised order, followed by measures assessing ED and OCD symptoms. Participants were also asked to report their age, height, weight, and ethnicity. After completing all of the measures, participants read a debriefing page providing further information about the study, and received credit on their Amazon account as compensation for their participation. This study was approved by the University’s ethics committee.

#### Locus of control

Locus of control was assessed using Levenson’s Internal, Powerful Others and Chance Scale (IPC) [29]. The IPC incorporates three subscales that measure the extent to which individuals believe that outcomes are due to their own actions (I: Internal Control), to powerful others (P: Powerful Others), or to chance or fate (C: Chance). Each subscale includes eight items that are rated on a 7-point Likert scale (−3 = *Strongly disagree*, +3 = *Strongly agree*), and items were summed and a constant of 24 added to each scale to eliminate negative sums. This measure has demonstrated moderate internal consistency and good construct validity [30]. For the current study, Cronbach’s alpha for each subscale was .70, .79 and .82, respectively.

#### Sense of control

The Shapiro Control Inventory (SCI) [31] measures domain-general and domain-specific perceived control, positive and negative control mechanism, and motivation for control. For this study, we focused on participants’ domain-general psychological sense of control by administering the 11-item positive sense of control scale (which measures perceived self-efficacy and self-control) and the 5-item negative sense of control scale (which captures loss of control, feelings of passivity and helplessness). A sample item from the positive sense of control scale (PSC) is, “If I decide to, I have the ability to make changes in order to gain more control over my life”. A sample item from the negative sense of control scale (NSC) is, “I lack control of my environment (other people, situations)”. For both subscales, each item is rated on a 6-point scale (1 = *Never*, 6 = *Very Often*). However, due to an administrative error, some of the items had 5 response options instead of 6, so scores were standardised and averaged. Both scales have yielded evidence of internal consistency and construct validity among clinical and normative populations [15, 31]. In the current study, Cronbach’s alpha was .90 for PSC and .76 for NSC.

#### Fear of losing self-control

Fear of losing self-control was measured using an adapted version of the Self-Control subscale of Reid and Ware’s Internal-External questionnaire [32]. Tiggemann and Raven [7] adapted the original 8-item scale to express fear of losing self-control by adding “I fear”, “I am afraid” or “I worry” to the beginning of each item. A sample item of the original scale is, “Sometimes I impulsively do things, which at other times I definitely would not let myself do”, and this item was changed to, “I worry that I sometimes will impulsively do things, which at other times I definitely would not let myself do.” Each item was rated on a 7-point scale that ranged from 1 (*Doesn’t apply to me at all*) to 7 (*Always applies to me*), with higher scores indicating greater doubts about being able to control one’s own impulses, desires, and emotional behaviour. This scale has been shown to be related to other measures of control and also to greater eating psychopathology [7]. Consistent with prior work [7], internal consistency was excellent in the current sample (Cronbach’s α = .94).

#### Desire for control

Burger and Cooper’s Desirability of Control Scale (DCS) [33] was administered to assess individual differences in general desire or need for control over life events. This measure contains 20 items with response options ranging from 1 (*This statement doesn’t apply to me at all*) to 7 (*This statement always applies to me*), and with higher scores indicating greater desire for control. A sample item is, “I try to avoid situations where someone else tells me what to do”. In previous studies, this measure has demonstrated evidence of internal consistency, test-retest reliability, and criterion validity [33]. For the current study, Cronbach’s alpha was .83.

#### Sense of mastery

Mastery was assessed using the Personal Mastery Scale developed by Pearlin and Schooler (PMS) [34]. This scale consists of seven items that are rated on a 5-point scale ranging from 1 (*Strongly disagree*) to 5 (*Strongly agree*). A sample item is, “I can do about anything I really set my mind to”. The total of the seven items was used as a measure of overall mastery, with higher scores reflecting greater personal mastery. For the current study, Cronbach’s alpha was .83.

#### Ineffectiveness

Participants completed the 10-item Ineffectiveness subscale of the Eating Disorder Inventory-2 (EDI-2-I) [35]. This subscale assesses feelings of general inadequacy, insecurity, worthlessness and lack of control over one’s life. Participants rate each item on a 6-point scale from 1(*Never*) to 5 (*Always*), with higher scores indicating a greater sense of ineffectiveness. High internal reliability estimates for the Ineffectiveness subscale have been demonstrated in both clinical and non-clinical samples [36, 37]. Consistent with previous research, Cronbach’s alpha in this study was high (α = .94).

#### Eating pathology

The Eating Disorder Diagnostic Scale (EDDS) [38] was used to assess ED psychopathology. This measure consists of 19 questions capturing ED symptoms, as described in 4^th^ edition of the *Diagnostic and Statistical Manual for Mental Disorders* (DSM–IV) [39]. The EDDS provides diagnoses for full threshold AN, full threshold BN, and full threshold Binge Eating Disorder (BED), as well as subthreshold AN, subthreshold BN, and subthreshold BED. Items can also be standardised and summed (excluding items asking about weight, height, and birth control pill use) to create a continuous ED symptom composite score (SCS). The current study utilised the EDDS diagnoses to delineate individuals who are free from ED symptoms versus those who are symptomatic, and the SCS to index participants’ overall level of eating pathology. Previous studies have yielded evidence of criterion validity, convergent validity, and internal consistency of the EDDS diagnoses and symptom composite [38, 40]. Cronbach’s alpha for the SCS in this study was .90.

#### Obsessive-compulsive symptoms

Participants completed the Obsessive–Compulsive Inventory - Revised (OCI-R) [41], an 18-item self-report measure of OCD and its various symptom presentations. The OCI-R has excellent reliability and validity and is widely used in clinical research [42]. Higher scores indicate a greater degree of obsessive-compulsive symptoms. Cronbach’s alpha in this study was .90.

### Statistical analyses

Prior to conducting the primary analyses, univariate and multivariate outliers were identified following the procedure described by Tabachnick and Fidell [43] and were excluded from all further analyses (*n* = 22). Univariate outliers were defined as those with *Z* scores > 3.00. Mahalanobis distance for the complete set of predictors was used to detect multivariate outliers; cases exceeding the critical chi-square value were omitted. The final sample consisted of 175 participants, and all analyses reported below are based on this final sample. The sample size was based on Tabachnick and Fidell’s [43] recommendation that a sample of N ≥ 104 + m is needed for adequate power in linear regression. With nine predictors included in the regression model, the sample size was more than adequate. All variables satisfied the assumptions of normality, linearity, homoscedasticity, and multicollinearity. Re-analysis of the data including outliers produced an identical pattern of results.

The first step in our analyses was to conduct bivariate correlations among all the variables included in the study. Next, in order to compare individuals high vs. low in psychopathology on the measures of control, we divided the sample into groups based on their scores on the EDDS and on the OCI-R. For the EDDS, we used the code developed by the scale’s authors to differentiate individuals meeting an ED diagnosis (full and subthreshold; ED group) from those who were symptom free (no-ED group). The ED group (*n* = 55; full diagnosis *n* = 34 and subthreshold diagnoses *n* = 21) consisted of 16 individuals with AN, 25 with BN, and 14 with BED. The remaining 120 participants did not fulfil one of the three main (or subthreshold) diagnoses. For the OCI-R, we utilised the cut-off score of 21 [41] to divide participants into those who scored high (OCD group; *n* = 39) and those who scored low on obsessive-compulsive symptoms (no-OCD group; *n* = 136). The high prevalence of psychopathology found in this study is consistent with recent work showing that MTurk participants endorse clinical symptoms to a substantially greater degree than do traditional nonclinical samples (e.g., undergraduate subject pools) [44]. Multivariate analyses of variance (MANOVA) was then used to examine significant differences between these groups on the dependent measures, with a Bonferroni correction applied to the follow-up univariate analyses. Although BMI was correlated with EDDS scores, the pattern of results did not change when controlling for participants’ BMI, and therefore BMI was not included in the analysis. Finally, we used multiple regression analyses to determine which measures of control were the strongest predictors of ED symptoms and OCD symptoms, separately. Because our predictors were all significantly correlated with one another (range = |.32| to |.74|), in addition to providing standardised regression weights, we also provided other indices of predictor importance that allow for more accurate variance partitioning among correlated predictors, including incremental R^2^, general dominance weights, and relative importance weights [45, 46].

## Results

### Correlation analyses

The bivariate correlations among all of the variables in this study are shown in Table 1, as are the means, standard deviations, and range for those variables. ED and OCD symptom severity were positively associated with external locus of control, negative sense of control, feelings of ineffectiveness, and fear of losing self-control, and were negatively associated with sense of mastery. Both eating pathology and obsessive-compulsive pathology were also negatively associated with internal locus of control, but the correlation with EDDS scores was not significant. Desire for control had a negligible zero-order correlation with both EDDS scores and OCI-R scores. It should also be noted that EDDS scores and OCI-R scores were positively correlated.

**Table 1:** Bivariate correlations, means, and standard deviations for all variables included in the analyses

	1	2	3	4	5	6	7	8	9	10	11	12
1. BMI	–											
2. LOC-I	−.07	–										
3. LOC-P	.05	−.46^***^	-									
4. LOC-C	.09	−.53^***^	.68^***^	–								
5. PSC	−.16^*^	.58^***^	−.48^***^	−.43^***^	–							
6. NSC	.20^**^	−.42^***^	.50^***^	.43^***^	−.67^***^	–						
7. FLC	.22^**^	−.39^***^	.51^***^	.45^***^	−.52^***^	.67^***^	–					
8. DCS	−.09	.37^***^	−.34^***^	−.32^***^	.53^***^	−.48^***^	−.36^***^	–				
9. PMS	−.13	.62^***^	−.53^***^	−.54^***^	.69^***^	−.66^***^	−.51^***^	.51^***^	–			
10. EDI-I	.24^**^	−.49^***^	.47^***^	.40^***^	−.73^***^	.71^***^	.58^***^	−.50^***^	−.74^***^	–		
11. SCS	.35^***^	−.13	.29^***^	.23^**^	−.26^**^	.32^***^	.50^***^	−.13	−.25^**^	.46^***^	–	
12. OCI-R	.14	−.26^***^	.24^**^	.18^*^	−.31^***^	.33^***^	.44^***^	−.06	−.30^***^	.36^***^	.37^***^	–
*M*	22.95	35.62	19.67	18.66	0	0	23.44	98.30	26.53	24.04	−.09	12.33
*SD*	6.81	5.97	8.58	8.78	0.70	0.72	10.43	14.09	4.46	9.76	0.55	10.30
*Min*	13.64	19	0	0	−2.02	−1.23	8	63	15	10	−.80	0
*Max*	45.77	48	42	43	1.35	2.14	52	137	35	51	1.16	41

*BMI* body mass index, *I-LOC* Locus of Control- Internal, *P-LOC* Powerful Others Locus of Control, *C-LOC* Chance Locus of Control, *PSC* Positive Sense of Control, *NSC* Negative Sense of Control, *FLC* Fear of Losing Self-Control, *DCS* Desirability of Control Scale, *PMS* Personal Mastery Scale, *EDI-I* Eating Disorder Inventory-Ineffectiveness, *SCS* Symptom Composite Score, *OCI-R* Obsessive-Compulsive Inventory-Revised

*p < .05. **p < .01. ***p < .001

### Multivariate analyses of variance

A MANOVA indicated a significant overall difference between the ED group and the no-ED group, *F* (9, 165) = 4.09, *p* < .001, η^2^_p_ = .18. Follow-up univariate analyses yielded significant group differences on five of the nine subscales (see Table 2). Participants in the ED group scored significantly higher than did those in the no-ED group on external LOC-powerful others, negative sense of control, ineffectiveness, and fear of losing self-control. They also reported significantly lower positive sense of control.

**Table 2:** Means (SD) and pairwise comparisons of each measure of control for EDDS and OCI-R groups

	EDDS				OCI-R			
	No-ED group	ED group	*p*-value^a^	η^2^_p_	No-OCD group	OCD group	*p*-value^a^	η^2^_p_
LOC-I	35.73 (6.26)	35.40 (5.33)	.74	.001	36.34 (5.76)	33.13 (6.07)	.003	.05
LOC-P	18.61 (8.47)	21.98 (8.43)	.02	.03	19.07 (8.85)	21.74 (7.29)	.09	.02
LOC-C	18.12 (8.97)	19.86 (8.30)	.23	.01	17.84 (8.93)	21.54 (7.66)	.02	.03
PSC	0.07 (0.70)	−0.16 (0.67)	.05	.02	0.08 (0.66)	−.29 (0.76)	.004	.05
NSC	−0.10 (0.69)	0.22 (0.74)	.01	.04	−.08 (0.68)	.27 (0.77)	.01	.04
FLC	20.90 (9.20)	28.98 (10.89)	< .001	.13	21.93 (9.84)	28.69 (10.86)	< .001	.07
DCS	98.25 (14.17)	98.40 (14.04)	.95	< .001	98.79 (14.24)	96.59 (13.58)	.39	.004
PMS	26.82 (4.33)	25.91 (4.74)	.21	.01	27.02 (4.27)	24.82 (4.75)	.01	.04
EDI-I	22.57 (8.76)	27.24 (11.09)	.003	.05	22.97 (9.46)	27.77 (10.03)	.01	.04

*I-LOC* Locus of Control- Internal, *P-LOC* Powerful Others Locus of Control, *C-LOC* Chance Locus of Control, *PSC* Positive Sense of Control, *NSC* Negative Sense of Control, *FLC* Fear of Losing Self-Control, *DCS* Desirability of Control Scale, *PMS* Personal Mastery Scale, *EDI-I* Eating Disorder Inventory-Ineffectiveness, *EDDS* Eating Disorder Diagnostic Scale, *OCI-R* Obsessive-Compulsive Inventory-Revised

^a^Adjusted by Bonferroni criteria for multiple comparisons

For the OCD groups, the MANOVA was also statistically significant, *F* (9, 165) = 2.12, *p* = .03, η^2^_p_ = .10, with seven of the nine measures of control yielding significant univariate differences between groups (Table 2). Notably, the pattern of results among the variables was quite similar between EDDS and OCI-R groups, with the OCD group scoring significantly lower than the no-OCD group on internal LOC, positive sense of control, and mastery, and scoring significantly higher on LOC-chance, negative sense of control, ineffectiveness, and fear of losing self-control.

### Regression analyses

Linear regressions analyses were conducted separately for EDDS SCS and OCI-R. For eating pathology, the overall model was significant, *F* (9, 165) = 10.36, *p* < .001, explaining 36 % of the variance in EDDS scores. Ineffectiveness and fear of losing self-control were the only significant independent predictors of EDDS scores (Table 3). To gain a broader and fuller perspective on the contributions that the control variables made to each regression equation, we also report incremental R^2^, general dominance weights, and relative importance weights. Consistent with the beta coefficients, ineffectiveness and fear of losing self-control were the strongest predictors of ED pathology across all three indices. They obtained the largest incremental R^2^, both on their own and when taking all other predictors into account. Dominance analysis results demonstrated complete dominance of ineffectiveness and fear of losing self-control over all other variables, as they contributed more unique variance in the regression effect across all possible model subsets in which they were included. Similarly, relative importance weights also favoured ineffectiveness and fear of losing self-control. Notably, the submodel containing only ineffectiveness and fear of loss of self-control accounted for 29 % of the variance. Therefore, by eliminating all variables except for ineffectiveness and fear of losing self-control, we are able to gain a more parsimonious model with very little loss in predictive efficiency (a loss of 7 %).

**Table 3:** Summary of statistics determining independent variable contributions to regression effect

	EDDS					OCI-R				
	*ß* weights	Incremental R^2^	Incremental R^2^_F_	DW	RIW	*ß* weights	Incremental R^2^	Incremental R^2^_F_	DW	RIW
LOC-I	.11	.02	.007	.008	.007	−.10	.07	.005	.018	.019
LOC-P	.05	.08	.001	.021	.022	.02	.06	< .001	.012	.012
LOC-C	.06	.05	.002	.012	.014	−.09	.03	.004	.007	.006
PSC	.10	.07	.004	.016	.019	−.06	.10	.001	.023	.022
NSC	−.15	.10	.007	.028	.029	−.01	.11	< .001	.028	.026
FLC	.44^***^	.25	.094	.144	.134	.37^***^	.19	.066	.101	.095
DCS	.09	.02	.005	.007	.007	.21^*^	.004	.031	.021	.014
PMS	.17	.06	.009	.018	.019	−.03	.09	< .001	.020	.019
EDI-I	.56^***^	.21	.099	.127	.109	.16	.13	.009	.041	.037

Entries are standardised coefficients. *R* ^*2*^_*F*_ Incremental R^2^ in full model, *DW* Dominance Weights, *RIW* Relative Importance Weights, *I-LOC* Locus of Control- Internal, *P-LOC* Powerful Others Locus of Control, *C-LOC* Chance Locus of Control, *PSC* Positive Sense of Control, *NSC* Negative Sense of Control, *FLC* Fear of Losing Self-Control, *DCS* Desirability of Control Scale, *PMS* Personal Mastery Scale, *EDI-I* Eating Disorder Inventory-Ineffectiveness

*p < .05

***p < .001

For OC symptoms, the overall model was also significant, *F* (9, 165) = 6.09, *p* < .001, explaining 25 % of the variance in OCI-R scores. Fear of losing self-control and desire for control emerged as the only significant independent predictors of OCI-R scores (Table 3). In this case, all three indices of predictor importance identified fear of losing self-control as the strongest contributor. By itself, fear of losing self-control explained 19.4 % of the variance of OCI-scores. Although desire for control had a statistically significant beta weight in the full regression model, it was not found to directly contribute variance to the dependent variable using other predictor importance measures.

## Discussion

The primary purpose of the present study was to elucidate how different control constructs relate to disordered eating behaviours. In particular, we examined the associations among sense of control, locus of control, fear of losing self-control, desire for control, mastery, ineffectiveness, and ED symptomatology. The results of this investigation provide further support for the role of control in disordered eating behaviours. When comparing women with high versus low levels of disordered eating behaviours, those with high scores on the EDDS were significantly more external in their locus of control, reported greater negative sense of control, feelings of ineffectiveness, and greater fear of losing self-control, as well as less positive sense of control. Analyses of relative predictor importance showed ineffectiveness and fear of losing self-control to be the variables that contributed the most to eating psychopathology. In fact, eliminating all other variables allowed for the most model parsimony without a substantial loss in variance explained.

The current findings suggest that, although a number of control dimensions are related to disordered eating behaviours, ineffectiveness and fear of losing self-control are the two strongest predictors of eating pathology. Ineffectiveness has received considerable research support in the ED literature [17, 47], and the present study adds to this growing body of evidence by suggesting that ineffectiveness may be critical to the development of disordered eating behaviours [48]. The second important dimension of control identified in this study, fear of losing self-control, has yet to be fully explored as a potential risk factor. Tiggemann and Raven [7] were first to report the importance of fear of losing self-control as a contributor to ED pathology when it emerged as the most potent predictor of ED symptomatology in their study. Surprisingly, this prominent result has not been further investigated and the measure of losing self-control developed by Tiggemann and Raven [7] is yet to be subjected to rigorous psychometric evaluation. The present study thus adds to the literature on the assessment of fear of losing self-control by providing additional evidence for the reliability and validity of the measure.

It is important to note that none of the items in any of the control scales administered in this study directly refer to eating, weight or shape. This suggests that the underlying control beliefs are not disorder-specific but are more general in nature, further supporting the “functional analysis” of ED [2]. The direction of causality between control and ED cannot be determined by the present result. We can speculate, however, that the need to control eating is a product of these individuals’ internalised sense of ineffectiveness and a fear of losing self-control. In response to feeling ill-prepared to function effectively in their lives, individuals with ED resort to auxiliary control mechanisms to ward off the fear of having no control at all [49]. In this respect, the illness can be understood as a functional response (albeit a maladaptive one) that derives from the failure of more appropriate mechanisms and sources of control [50].

The secondary aim of this research was to explore whether the constructs of control that are relevant to ED are also related to OCD symptoms. The results revealed a similar pattern to that observed with eating pathology. In comparison to the group with low OCI-R scores, women with high scores displayed significantly more externality, negative sense of control, feelings of ineffectiveness, fear of losing self-control, and less sense of mastery, positive sense of control, and internal LOC. Relative importance analysis identified fear of losing self-control to be the most important predictor of OCI-R scores. It is interesting to note that desire for control was not related to OCD symptoms in the bivariate correlations, but was a significant predictor in the regression model. This pattern of results has been reported previously [28] and suggested a possible suppression effect. Overall, the results support the contention that both disorders may have similar underlying issues of control. On the one hand, the findings suggest that both disorders are characterised by a fear of losing self-control. This is consistent with other studies that found marked fear of losing control over mental activities, impulses, and emotions in patients with OCD [51]. On the other hand, feelings of inadequacy, worthlessness and lack of control, as measured by the EDI-Ineffectiveness scale, seem to be aspects that are especially characteristic of ED [52].

The results of the present study should be interpreted in the context of their limitations. First, we conducted the study with a female, non-clinical sample and so the results await replication in males and clinical samples. However, research on non-clinical populations in this domain is important given the prevalence of disordered eating in today’s society [53] and the potential for such research to contribute to prevention and intervention efforts. Furthermore, research suggests that obsessive-compulsive symptoms in non-clinical community samples are qualitatively indistinguishable from those in clinically diagnosed samples of OCD patients [54], thus supporting the assumption that obsessive-compulsive symptoms and dysfunctional beliefs are dimensional rather than categorical in nature. It therefore seems likely that the associations found in the current study between issues of control and ED/OCD symptoms would be similar, if not stronger, among individuals with symptoms severe enough to meet the criteria for a diagnosis. Second, another limitation of the present study was the exclusive reliance on self-reports for assessment. Future research is needed to verify the results of this study using more diverse and reliable methods. In particular, the use of interview methods to arrive at a clear-cut diagnosis of ED is advocated. Third, the present study did not explore whether different control measures differentially relate to different ED diagnoses or symptoms. Future research should explore this possibility either by including measures that capture diagnosis-specific symptoms or by conducting this study with a clinical sample that provides sufficiently large diagnostic groups to ensure adequate statistical power. Finally, the present study sought to distinguish among existing measures of control. It is possible that some measures of control include multiple dimensions of control within the same measure, or that there are higher order constructs that would be apparent across measures. A step forward for researchers would be to collect data on a much larger sample, submit all items simultaneously to a joint exploratory factor analysis, and reanalyse the relationships between the newly created factors and ED/OCD symptoms.

## Conclusions

The findings provide some evidence that ineffectiveness and fear of losing self-control are two dimensions that are important to consider in maintenance and treatment models of disordered eating behaviours. Treatment approaches that solely focus on stringent behavioural goals (i.e., reduce control over eating, weight, and shape) may fail to address the deeper problem underlying and often maintaining these symptoms [55]. Helping the individual re-established adaptive mechanisms of personal control and effectiveness may reduce their need to rely on weight/shape control. Similarly, individuals vulnerable to OCD may benefit from psychotherapeutic interventions that challenge worries of losing control over one’s impulses, desires and emotional behaviours (i.e., self-control). Future work should examine how these cognitions can be targeted in prevention and intervention efforts.

## Ethics

All procedures performed in studies involving human participants were in accordance with the ethical standards of a medical ethics committee and with the 1964 Helsinki declaration and its later amendments or comparable ethical standards. This project (HREAP 143–132) has been approved by the Human Research Ethics Advisory Panel for the School of Psychology, UNSW.

## Informed consent

Informed consent was obtained from all individual participants included in the study.
